# EDUCATION AND TRAINING II: AGE-INCLUSIVITY AND HEALTH PROMOTION APPROACHES

**DOI:** 10.1093/geroni/igae098.0466

**Published:** 2024-12-31

## Abstract

Cognition/ADRD; Family/social Aspects; Community-based approaches

